# Investigation of altering single-nucleotide polymorphism density on the power to detect trait loci and frequency of false positive in nonparametric linkage analyses of qualitative traits

**DOI:** 10.1186/1471-2156-6-S1-S20

**Published:** 2005-12-30

**Authors:** Alison P Klein, Ya-Yu Tsai, Priya Duggal, Elizabeth M Gillanders, Michael Barnhart, Rasika A Mathias, Ian P Dusenberry, Amy Turiff, Peter S Chines, Janet Goldstein, Robert Wojciechowski, Wayne Hening, Elizabeth W Pugh, Joan E Bailey-Wilson

**Affiliations:** 1 Inherited Disease Research Branch, NHGRI/NIH, Baltimore, MD, USA; 2 Sidney Kimmel Comprehensive Cancer Center, Johns Hopkins School of Medicine, Baltimore, MD, USA; 3 CIDR, Johns Hopkins Medical School, Baltimore, MD, USA; 4 Genome Technology Branch, NHGRI/NIH, Bethesda, MD, USA; 5 Department of Neurology, UMDNJ-RW Johnson Medical School, New Brunswick, NJ, USA

## Abstract

Genome-wide linkage analysis using microsatellite markers has been successful in the identification of numerous Mendelian and complex disease loci. The recent availability of high-density single-nucleotide polymorphism (SNP) maps provides a potentially more powerful option. Using the simulated and Collaborative Study on the Genetics of Alcoholism (COGA) datasets from the Genetics Analysis Workshop 14 (GAW14), we examined how altering the density of SNP marker sets impacted the overall information content, the power to detect trait loci, and the number of false positive results. For the simulated data we used SNP maps with density of 0.3 cM, 1 cM, 2 cM, and 3 cM. For the COGA data we combined the marker sets from Illumina and Affymetrix to create a map with average density of 0.25 cM and then, using a sub-sample of these markers, created maps with density of 0.3 cM, 0.6 cM, 1 cM, 2 cM, and 3 cM. For each marker set, multipoint linkage analysis using MERLIN was performed for both dominant and recessive traits derived from marker loci. Our results showed that information content increased with increased map density. For the homogeneous, completely penetrant traits we created, there was only a modest difference in ability to detect trait loci. Additionally, as map density increased there was only a slight increase in the number of false positive results when there was linkage disequilibrium (LD) between markers. The presence of LD between markers may have led to an increased number of false positive regions but no clear relationship between regions of high LD and locations of false positive linkage signals was observed.

## Background

Genome-wide linkage analysis using microsatellite markers has been successful in the identification of numerous Mendelian and complex disease loci. Recently available high-density single-nucleotide polymorphism (SNP) maps theoretically provide greater information content (IC), which should help to both identify and narrow linkage regions. This is supported by a few published reports comparing genome-wide linkage analysis using microsatellites to studies of the same dataset using dense SNP maps [1,2]. Yet questions remain about the optimal density of SNP marker sets for linkage studies. Additionally, current algorithms for linkage analysis assume that adjacent markers are in linkage equilibrium. However, there may be significant linkage disequilibrium (LD) between adjacent markers in dense SNP marker sets, which can lead to false positive results [3]. To explore these issues we used the simulated and Collaborative Study on the Genetics of Alcoholism (COGA) datasets to examine how altering the SNP density impacted the overall IC, the power to detect trait loci, and the number of false positive results. We compared these results to analyses performed using microsatellite markers.

## Methods

### Simulated data

Analyses were performed (separately for each population and replicate) using all replicates of the Aiputo, Danaca, and Karanga populations. The full marker sets for both the MS (7.5 cM) and SNP (3 cM) maps were used. Additional fine mapping markers were purchased for chromosomes 8 and 9 (packets 400–406 and 416–419) to increase the density of the SNPs (0.3 cM). We had knowledge of the answers.

### Trait definition (simulated)

Dominant or recessive traits were created using these marker loci: B08T8044, B08T8045, B08T8050, and B08T8051. Affection status for a dominant trait was defined as individuals with ≥ 1 copy of allele 1 at the marker and for a recessive trait as individuals with 2 copies of allele 1.

### COGA data

Using a perl script, we created an interpolated genetic map that used MS markers from the deCode map and SNPs from both Illumina and Affymetrix. For each SNP, 2 MS markers from the deCode map were identified that flanked the SNP using the physical positions of these markers obtained from sequence build 34. From the physical and genetic position of the 2 flanking microsatellites and assuming a linear interpolation between the markers, the genetic position of the SNP was determined. Any MS or SNP without a physical position was removed. If SNP markers mapped to the same genetic location, the SNP with the largest physical location was kept.

### Trait definition (COGA)

The following markers (and risk alleles) were used to create a dominant and/or a recessive trait: rs0041510 (allele 2), tsc2832191 (allele 1), tsc0061481 (allele 1). To avoid errors due to differences in allele frequencies between ethnic groups, analysis was limited to the white/non-Hispanic families, which comprised the largest ethnic subgroup.

### Creation of SNP maps

Using a perl script, we selected a subset of the SNP markers to create maps that were less dense. Our goal was to select markers with desired inter-marker distances. To avoid tight clusters of markers, we moved at least the desired distance minus 10% of that distance before another marker was selected. If there were multiple markers within ± 10% of the desired distance, the marker with the major allele frequency (MAF) closest to 0.5 was selected. For example, for the 0.3-cM map, markers were forced to be at least 0.27 cM apart, and if there were multiple markers located between 0.27 cM and 0.33 cM from the last marker, the marker with the MAF closest to 0.5 was selected.

### Statistical analysis

We used the analysis program MERLIN for all linkage analyses [4]. Allele frequencies were estimated from all founders. Kong and Cox LOD scores [5] and the associated *p*-values for Whittemore and Halpern's NPL_All _[6] statistic were used for the analysis of qualitative traits. Entropy, a measure of IC, was used. Multipoint evaluation was performed at each of the marker loci (between-marker evaluations were not performed). For the evaluation of power and type I error we used 4 standard *p*-value thresholds (0.05, 0.01, 0.001, and 0.0001) and 2 Lander-Krugylak [7] genome-wide significance levels. We calculated power as the number of replicates with a *p*-value less than the threshold within a 20 cM region (10 cM in either direction) of the trait loci. To assess the frequency of false positive results, we counted the number of regions where a *p*-value less than the above-mentioned cut-off occurred on chromosomes not containing the trait loci. In order to ensure that adjacent makers with *p*-values below the given level were not counted as multiple false positive results, a region with a *p*-value greater than or equal to 0.2 was required to occur between two false positive regions.

## Results

Table 1 presents the results of our comparison of the IC for the various map densities. In the simulated data, the average IC of the MS map was 0.934. There is a loss in information when we compared the 3-cM SNP map (0.833) to the MS map. Conversely, a very dense SNP map showed a modest increase in IC (0.986); the mean IC was highest in the very dense (0.3 cM) SNP map (0.986). In the COGA dataset IC increased with increasing map density and was lowest in the MS marker set. The overall IC was a bit lower in the COGA data; this could be due in part to the presence of missing data in the COGA dataset or overall marker heterozygosity. Note that the MS map in the COGA dataset (13.6 cM) is less dense than the MS map in the simulated dataset (7.5 cM).

There was a modest increase in power with increasing SNP map density in the simulated data (Table 2). Power was greatest for the 0.3-cM density. Power for the MS map seemed to fall between the 1 cM and 3 cM SNP map. Overall power was quite low when we used a genome-wide significance level of 0.000049. However, in the COGA dataset (Table 3) there were less consistent trends in the ability to detect the trait loci as map density increased. In fact, the denser maps sometimes gave smaller LOD scores as compared with less dense maps (e.g., Drs0041510). This could be due to errors in marker order or inter-marker distance for the denser map sets. It is important to note that our created traits were homogenous and had complete penetrance, and thus overall power was very high, possibility masking any true variations in power due to differences in map density. For all map sets disease frequencies had a large impact on power. Additionally, given we only performed analysis at the marker loci and not between marker loci, we cannot evaluate if denser maps yielded smaller confidence intervals for the linkage peaks because 1-LOD confidence intervals are dependant upon the density of analytic evaluations.

The number of false positive linkages (*p*-value below a given level in a region unlinked to the trait loci) for the simulated data is in Tables 4 and 5. When we compare the results for the 3-cM SNP map to the MS map or the 0.3 cM to the 1-cM SNP map, the number of false positive results remains similar. Although the 0.3-cM map has a slight increase in the number of false positive results compared to the 1-cM map, it is hard to interpret this because such a dense map was only available in one 18-cM region. We also examined the number of false positive regions for each of the traits in the COGA dataset (Table 6) by tabulating significant linkages on 18 unlinked chromosomes. Overall, the number of false positive regions at the 0.05 level was greater in the combined 0.25-cM SNP map than it was in the less dense maps. At the more stringent *p*-value levels there were only a few false positive results, and no false positives were observed for any of the traits at genome-wide significant *p*-values (0.000049) [7].

## Conclusion

Overall, IC was higher for the dense SNP maps as compared with the less dense SNP and MS maps. In the simulated data, there was a modest increase in power with increasing SNP map density. However in the COGA data, no consistent trends were observed in our ability to detect trait loci with increasing map density. There was variation in the LOD scores across maps, with more dense maps sometimes yielding lower LOD scores. This could be due to errors in map order and supports the need for precise genetic maps when using dense SNP maps for linkage. Unsurprisingly, power was dependent on disease prevalence for these homogeneous, completely penetrant traits.

In the simulated data, in which there was no significant LD between markers, the number of false positives did not increase with increasing map density. In the COGA data, more false positives were observed for the densest map set, 0.25 cM, in which there was significant intermarker LD. Huang et al. [3] reported that the presence of intermarker LD caused an increase in false positives, particularly when there is missing parental data. This is of particular concern because others have reported that SNPs are more powerful than microsatellites when there is missing parental data. To examine this, we calculated the LD between all SNPs up to 500 kb apart. Twenty-one percent of all pairwise SNPs had a D' > 0.70 (high LD). Of those SNPs with a D' > 0.70, 89% were <200 kb apart, 9% were 200–400 kb apart and 2% >400 kb apart. The LD between SNPs diminished as distance increased, suggesting maps with an average marker distance >200 kb would have limited intermarker LD. Comprehensive review of the locations of all type I errors observed for two of these traits (created from marker tsc006148 on chromosome 13) showed that while 90% of these regions contained markers exhibiting LD, the LD patterns in these regions did not differ markedly from the LD on the remainder of the chromosomes. Interestingly, 20% of the false positives occurred at the telomeres of chromosomes. While some of the increases in numbers of type I errors could be due to increased intermarker LD in the densest maps, they could also be caused by the fact that more evaluations of linkage were performed for the dense maps, since we evaluated linkage at each marker location and did not perform any intermarker evaluations. Thus, the densest map had the largest number of linkage tests performed (see Table 1), so increased type I errors could be due to LD or to increased tests.

## Abbreviations

COGA: Collaborative Study of the Genetics of Alcoholism

GAW14: Genetic Analysis Workshop 14

IC: Information content

LD: Linkage disequilibrium

MAF: Major allele frequency

MS: Microsatellite

SNP: Single-nucleotide polymorphism

## Figures and Tables

**Table 1:** Information content

	Marker set	Number of marker in map	Mean minimum^a ^(SD)	Overall mean^a ^(SD)	Mean maximum^a ^(SD)
Simulated	MS (~7.5 cM)	416	0.812 (0.077)	0.934 (0.004)	0.9724 (0.005)
	SNP 3 cM	917	0.644 (0.084)	0.833 (0.015)	0.914 (0.010)
	SNP 1 cM^b^	34	0.849 (0.018)	0.937 (0.006)	0.969 (0.006)
	SNP 0.3 cM^b^	201	0.933 (0.013)	0.986 (0.001)	0.998 (0.001)
COGA	MS (~13.5 cM)	315	0.586 (0.078)	0.744 (0.060)	0.840 (0.064)
	SNP 3 cM	1103	0.674 (0.076)	0.747 (0.012)	0.820 (0.015)
	SNP 2 cM	1792	0.566 (0.074)	0.767 (0.010)	0.825 (0.010)
	SNP 1 cM	2382	0.692 (0.055)	0.868 (0.008)	0.910 (0.007)
	SNP 0.6 cM	3671	0.724 (0.059)	0.895 (0.006)	0.930 (0.006)
	SNP 0.3 cM	5405	0.751 (0.062)	0.916 (0.005)	0.943 (0.006)
	SNP 0.25 cM	15015	0.825 (0.046)	0.939 (0.005)	0.955 (0.003)

^a^Overall mean, average minimum, and average maximum information content across all 3 populations and replicates for simulated data and across all 22 chromosomes for COGA data.

^b^The SNP 1 cM and SNP 0.3 cM map for the simulated data are based only on the regions for which fine mapping markers were purchased.

**Table 2:** Power in simulated data

Trait	Marker set	Pop. dz. freq.	Percentage of replicates with *p*-value below^a^					
			
			0.05	0.01	0.0017	0.001	0.0001	0.000049
Dominant								
D8044	MS 7.5 cM	0.06	0.96	0.89	0.67	0.57	0.27	0.16
	SNP 3 cM		0.95	0.86	0.60	0.51	0.18	0.12
	SNP 1 cM		0.98	0.90	0.72	0.63	0.29	0.21
	SNP 0.3 cM		0.96	0.92	0.77	0.69	0.37	0.27
								
D8050	MS 7.5 cM	0.18	1.00	1.00	0.99	0.99	0.97	0.94
	SNP 3 cM		1.00	1.00	0.99	0.99	0.93	0.91
	SNP 1 cM		1.00	1.00	1.00	1.00	0.98	0.98
	SNP 0.3 cM		0.99	0.99	0.99	0.99	0.97	0.96
								
D8051	MS 7.5 cM	0.50	0.99	0.95	0.88	0.83	0.62	0.54
	SNP 3 cM		0.99	0.95	0.85	0.81	0.59	0.51
	SNP 1 cM		1.00	0.97	0.90	0.88	0.70	0.61
	SNP 0.3 cM		1.00	0.98	0.92	0.9	0.77	0.70
								
Recessive								
R8045	MS 7.5 cM	0.08	0.99	0.93	0.74	0.67	0.32	0.22
	SNP 3 cM		0.98	0.89	0.69	0.61	0.24	0.15
	SNP 1 cM		0.99	0.95	0.78	0.73	0.36	0.26
	SNP 0.3 cM		0.98	0.95	0.84	0.78	0.44	0.34
								
R8050	MS 7.5 cM	0.01	0.46	0.10	0.005	0	0	0
	SNP 3 cM		0.41	0.05	0	0	0	0
	SNP 1 cM		0.79	0.45	0.13	0.004	0	0
	SNP 0.3 cM		0.47	0.13	0.005	0.005	0	0
								
R8051	MS 7.5 cM	0.22	1.00	1.00	0.96	0.94	0.83	0.78
	SNP 3 cM		1.00	1.00	0.94	0.92	0.81	0.74
	SNP 1 cM		1.00	1.00	0.98	0.98	0.88	0.85
	SNP 0.3 cM		0.99	0.99	0.99	0.98	0.90	0.88

^a^Percentage of replicates with *p*-value below the following criteria within a 20 cM range of the given "true" trait locus. The results were summarized across the 3 simulated populations. Each population was analyzed separately.

**Table 3:** Power in COGA data

Trait	Marker set	Dz. freq.	LOD	Minimum *P*-value^a^
Dominant				
Drs0041510	MS	0.14	3.1	0.00008
	SNP 3 cM		3.8	0.00001
	SNP 2 cM		2.8	0.0002
	SNP 1 cM		4.1	0.00001
	SNP 0.6 cM		3.0	0.00008
	SNP 0.3 cM		3.9	0.00001
	SNP 0.25		4.1	0.00001
Dtsc0061481	MS	0.31	1.0	0.02^b^
	SNP 3 cM		5.7	<0.00001
	SNP 2 cM		5.0	<0.00001
	SNP 1 cM		5.9	<0.00001
	SNP 0.6 cM		6.1	<0.00001
	SNP 0.3 cM		6.3	<0.00001
	SNP 0.25		6.1	<0.00001
Recessive				
Rtsc0061481	MS	0.03	0.81	0.03^b^
	SNP 3 cM		1.65	0.003
	SNP 2 cM		1.70	0.003
	SNP 1 cM		1.68	0.002
	SNP 0.6 cM		1.68	0.003
	SNP 0.3 cM		1.68	0.002
	SNP 0.25		1.79	0.002
Rtsc2832191	MS	0.22	6.0	<0.00001
	SNP 3 cM		4.5	<0.00001
	SNP 2 cM		6.2	<0.00001
	SNP 1 cM		6.7	<0.00001
	SNP 0.6 cM		7.4	<0.00001
	SNP 0.3 cM		7.5	<0.00001
	SNP 0.25		3.6	<0.00003

^a^Minimum *p*-value within 20 cM of the "true" trait locus

^b^Marker D13S325 located about 12.3 cM from the trait loci gave a *p*-value of 0.0004 for trait Dtsc0061481 and a *p*-value of 0.004 for trait Rtsc0061481

**Table 4:** Type I error count in simulated data for full dataset

Trait	Marker set	Pop. dz. freq.	# of replicates with data^a^	Mean number of false positives below *p*-value criterion of^b^					
				
				0.05	0.01	0.0017	0.001	0.0001	0.000049
Dominant									
D8044	MS 7.5 cM	0.06	300	8.10	1.92	0.18	0.08	0	0
	SNP 3 cM			7.73	1.79	0.16	0.09	0	0
D8050	MS 7.5 cM	0.18	300	7.63	1.82	0.53	0.38	0.03	0.02
	SNP 3 cM			7.33	2.11	0.47	0.30	0.03	0.02
D8051	MS 7.5 cM	0.50	300	7.48	2.56	0.45	0.30	0.04	0.02
	SNP 3 cM			7.22	2.19	0.45	0.30	0.04	0.02
Recessive									
R8045	MS 7.5 cM	0.08	300	8.59	2.36	0.29	0.16	0.01	0
	SNP 3 cM			8.11	2.23	0.25	0.14	0.01	0
R8050	MS 7.5 cM	0.01	229	2.66	0.06	0	0	0	0
	SNP 3 cM			2.20	0.03	0	0	0	0
R8051	MS 7.5 cM	0.22	300	7.57	2.02	0.39	0.27	0.03	0.02
	SNP 3 cM			7.44	2.03	0.47	0.34	0.04	0.01

^a^For rare disease not all replicates contained informative pedigrees.

^b^Mean number of false positive regions in the 9 unlinked chromosomes per replicate with *p-*value below the following criteria.

**Table 5:** Type I error count in densely mapped simulated data

Trait	Marker set	Pop. dz. freq.	# of replicates with data	Mean number of false positives below *p*-value criterion of^a^					
				
				0.05	0.01	0.0017	0.001	0.0001	0.000049
Dominant									
D8044	SNP 1 cM	0.06	300	0.157	0.063	0	0	0	0
	SNP 0.3 cM			0.177	0.053	0.010	0.007	0	0
D8050	SNP 1 cM	0.18	300	0.146	0.003	0.007	0.007	0.003	0
	SNP 0.3 cM			0.18	0.037	0.013	0.007	0.003	0
D8051	SNP 1 cM	0.50	300	0.230	0.057	0.02	0.013	0	0
	SNP 0.3 cM			0.280	0.100	0.02	0.017	0	0
Recessive									
R8045	SNP 1 cM	0.08	300	0.113	0.037	0.007	0.003	0	0
	SNP 0.3 cM			0.147	0.050	0.003	0	0	0
R8050	SNP 1 cM	0.01	229	0.039	0.004	0	0	0	0
	SNP 0.3 cM			0.037	0.004	0	0	0	0
R8051	SNP 1 cM	0.22	300	0.160	0.060	0.020	0.020	0.007	0.007
	SNP 0.3 cM			0.183	0.063	0.020	0.003	0.007	0.007

^a^Mean number of false positive results in the ~18 cM unlinked region per replicate with *p*-value below the following criteria.

**Table 6:** Type I error in COGA data

Trait	SNP Set	Dz. freq.	Number of false positive below *p*-value criterion of^a^					
			
			0.05	0.01	0.0017	0.001	0.0001	0.000049
Dominant								
Drs0041510	MS	0.14	7	2	2	2	0	0
	SNP 3 cM		10	3	1	0	0	0
	SNP 2 cM		7	1	0	0	0	0
	SNP 1 cM		15	2	1	1	0	0
	SNP 0.6 cM		12	5	1	1	0	0
	SNP 0.3 cM		14	5	2	1	0	0
	SNP 0.25		18	8	2	1	0	0
Dtsc0061481	MS	0.31	6	1	1	0	0	0
	SNP 3 cM		8	4	2	1	0	0
	SNP 2 cM		12	6	2	1	0	0
	SNP 1 cM		17	7	0	0	0	0
	SNP 0.6 cM		16	6	2	2	0	0
	SNP 0.3 cM		15	7	1	1	0	0
	SNP 0.25		24	9	3	1	0	0
Recessive								
Rtsc0061581	MS	0.03	8	0	0	0	0	0
	SNP 3 cM		7	0	0	0	0	0
	SNP 2 cM		7	0	0	0	0	0
	SNP 1 cM		9	0	0	0	0	0
	SNP 0.6 cM		10	0	0	0	0	0
	SNP 0.3 cM		9	0	0	0	0	0
	SNP 0.25		13	1	0	0	0	0
Rtsc2832191	MS	0.22	6	4	2	0	0	0
	SNP 3 cM		10	3	1	0	0	0
	SNP 2 cM		12	3	2	2	0	0
	SNP 1 cM		13	4	1	1	0	0
	SNP 0.6 cM		14	6	2	1	0	0
	SNP 0.3 cM		16	6	1	1	1	0
	SNP 0.25		23	6	2	2	0	0

^a^Number of false positive regions across the 18 unlinked chromosomes with *p*-value below the following criteria.
